# The Availability and Price of Healthy Food in Seattle by Neighborhood Sociodemographic Characteristics

**DOI:** 10.5888/pcd19.220035

**Published:** 2022-11-23

**Authors:** Leah Neff Warner, Lina Pinero Walkinshaw, Vanessa M. Oddo, Melissa A. Knox, Philip M. Hurvitz, Anita Rocha, Nadine Chan, Brian E. Saelens, Jessica C. Jones-Smith

**Affiliations:** 1 University of Washington, School of Public Health, Department of Epidemiology, Seattle, Washington; 2 University of Washington, School of Public Health, Department of Health Systems and Population Health, Seattle, Washington; 3 University of Illinois Chicago, College of Applied Health Sciences, Department of Kinesiology and Nutrition, Chicago, Illinois; 4 University of Washington, Department of Economics, Seattle, Washington; 5 University of Washington, Urban Form Lab, Department of Urban Design and Planning, Seattle, Washington; 6 University of Washington, Center for Studies in Demography and Ecology, Seattle, Washington; 7 Washington State Department of Social and Health Services, Olympia, Washington; 8 Public Health, Seattle & King County, Assessment, Policy, Development and Evaluation Division, Seattle, Washington; 9 Seattle Children’s Research Institute, Seattle, Washington; 10 University of Washington, School of Medicine, Department of Pediatrics, Seattle, Washington

## Abstract

**Introduction:**

Unequal access to healthy food in the local food retail environment contributes to diet quality disparities. We assessed whether in-store availability and prices of healthy foods differ by neighborhood-level income and racial and ethnic composition in a representative sample of food stores in Seattle, Washington.

**Methods:**

We developed and validated an in-store survey tool and surveyed 134 stores. We measured availability and prices of 19 items. For each store, we calculated a healthy food availability score (range, 0–25), and mean prices within each category. Using census tract data, we identified the median household income and proportions of Black and Hispanic residents for each store’s neighborhood and grouped them by tertiles of these neighborhood characteristics across Seattle census tracts. We used Wald tests to compare mean availability scores and prices between tertiles and applied postestimation weights to reflect store-type distributions within each tertile.

**Results:**

Neighborhoods with lower income and a larger proportion of Black residents had lower healthy food availability scores compared with neighborhoods with higher income (8.06 [95% CI, 7.04–9.07] vs 12.40 [95% CI, 10.63–14.17], *P < .*001) and fewer Black residents (8.88 [95% CI, 7.79–9.98] vs 12.32 [95% CI, 10.51–14.14], *P = .*003). Availability did not differ by Hispanic population proportions. Mean prices of grains, eggs, and meat were lower in neighborhoods with larger proportions of Black residents.

**Conclusion:**

We found systematic differences in healthy food availability based on neighborhood-level income and racial composition. In-store assessments of the food retail environment can inform local, tailored strategies to improve healthy food access.

**Summary**

**What is already known on this topic?**

Unequal access to healthy foods may contribute to diet quality disparities. Systematic differences exist in the mix of store types by neighborhood-level income and racial and ethnic composition. Healthy food availability and prices vary between store types.

**What is added by this report?**

An abbreviated in-store survey tool is a valid assessment of healthy food availability in the local food environment. In a representative sample of Seattle stores, in-store availability of healthy food differed by neighborhood-level income and proportion of Black residents.

**What are the implications for public health practice?**

Local assessments of the food retail environment can inform tailored strategies to increase healthy food availability and affordability in target areas.

## Introduction

Multiple dimensions of food access, including availability, accessibility (ie, geographic proximity), affordability, accommodation, and acceptability, are important to achieving healthy diet quality (1). Neighborhood-level systematic differences in the food environment, largely measured through geographic access to food retail establishments, have been documented (2–4). Lower-income neighborhoods are more likely to have more convenience stores and fewer supermarkets than higher-income neighborhoods (5,6). Studies have also found racial and ethnic disparities whereby communities of color have less access to supermarkets and more access to convenience stores and fast-food restaurants compared with majority-White neighborhoods (2,5,7–9). Unequal access to different types of stores is important because store type is correlated with food healthfulness, availability, and price (5,6,10–12).

Limitations of relying solely on the presence or absence of types of food stores to evaluate healthy food access are increasingly recognized. Wide heterogeneity exists in the offering of healthy foods in medium and small stores; supermarkets are also a source of unhealthy food items (1,13). Few studies have investigated disparities in in-store availability and prices (10,12–17). Much of the research examining neighborhood-level disparities has focused on limited types of food stores (13,14,16), and few have addressed neighborhood-level (rather than city-level) differences across a range of food retailers (10,12,15). Additionally, few studies have used validated measurement tools to assess food availability (1,10,12).

In addition to limited evidence on in-store availability, it is unclear whether in-store food prices differ by neighborhood sociodemographic characteristics (13,18). Limited research has examined whether neighborhood characteristics are related to the economic food environment beyond known differences in store type distribution (13,19). In a study of food prices in US supermarkets, prices of healthy foods did not differ by neighborhood socioeconomic status or neighborhood racial and ethnic composition (16). A study of Seattle supermarkets suggested that shoppers may select stores on the basis of average food prices rather than geographic proximity (11). Neighborhood-level pricing patterns in other store types have not been thoroughly examined.

Multidimensional assessments of healthy food access within stores of varying types are needed to better understand neighborhood-level disparities in the food environment. Our primary objective was to assess in-store 1) availability and 2) prices of healthy foods in Seattle, Washington, by neighborhood sociodemographic characteristics through the development, validation, and application of an abbreviated measurement tool for in-store retail audits. We examined availability and prices by neighborhood-level measures of household income and racial and ethnic composition to assess whether systematic differences in healthy food access exist for these neighborhood factors.

## Methods

### Study design and sample

We developed and validated an abbreviated in-store measurement tool, the Seattle Healthy Food Survey, to conduct a cross-sectional assessment of healthy food availability and prices in Seattle, Washington. We included a geographically balanced sample of 134 food stores classified by store type, including supermarkets, warehouses/superstores, grocery stores, small stores, and drug stores (Table 1). Trained data collectors completed in-store assessments between May 21 and July 20, 2018.

**Table 1:** Comparison of Seattle Food Stores in the 2015 Categorized Food Permit Database to the 2018 Seattle Healthy Food Survey Store Sample, Seattle, Washington, 2018^a^

Characteristic	All Seattle stores in categorized food permit database (N = 493)	Store sample (n = 134)	Percentage point difference in proportion (food permit database minus store sample),^b^
	No. (%)		
**Store type**			
Total in Seattle	493 (100.0)	134 (100.0)	0
Supermarket	58 (11.8)	23 (17.2)	−5.4
Warehouse/superstore	7 (1.4)	7 (5.2)	−3.8
Grocery	86 (17.4)	29 (21.6)	−4.2
Small store	298 (60.5)	58 (43.3)	17.2
Drug store	44 (8.9)	17 (12.7)	−3.8
**Median household income in census tract**			
$10,865–$65,772 (1st tertile^c^)	226 (45.8)	62 (46.3)	−0.5
Supermarket	20 (8.9)	10 (16.1)	−7.2
Warehouse/superstore	3 (1.3)	3 (4.8)	−3.5
Grocery	34 (15.0)	12 (19.4)	−4.4
Small store	151 (66.8)	28 (45.2)	21.6
Drug store	18 (8.0)	9 (14.5)	−6.5
$65,781–$90,688 (2nd tertile)	183 (37.1)	46 (34.3)	2.8
Supermarket	27 (14.8)	9 (19.6)	−4.8
Warehouse/superstore	4 (2.2)	4 (8.7)	−6.5
Grocery	30 (16.4)	9 (19.6)	−3.2
Small store	103 (56.3)	18 (39.1)	17.2
Drug store	19 (10.4)	6 (13.0)	−2.6
$90,855–$159,652 (3rd tertile)	84 (17.0)	26 (19.4)	−2.4
Supermarket	11 (13.1)	4 (15.4)	−2.3
Warehouse/superstore	0	0	0
Grocery	22 (26.2)	8 (30.8)	−4.6
Small store	44 (52.4)	12 (46.2)	6.2
Drug store	7 (8.3)	2 (7.7)	0.6
**Percentage of Black population in census tract**			
0%–1.41% (1st tertile)	83 (16.8)	28 (20.9)	−4.1
Supermarket	15 (18.1)	6 (21.4)	−3.3
Warehouse/superstore	0	0	0
Grocery	15 (18.1)	6 (21.4)	−3.3
Small store	46 (55.4)	12 (42.9)	12.5
Drug store	7 (8.4)	4 (14.3)	−5.9
1.43%–7.17% (2nd tertile)	173 (35.1)	37 (27.6)	7.5
Supermarket	22 (12.7)	6 (16.2)	−3.5
Warehouse/superstore	2 (1.2)	2 (5.4)	−4.2
Grocery	35 (20.2)	9 (24.3)	−4.1
Small store	95 (54.9)	17 (46.0)	8.9
Drug store	19 (11.0)	3 (8.1)	2.9
7.31%–40.01% (3rd tertile)	237 (48.1)	69 (51.5)	−3.4
Supermarket	21 (8.9)	11 (15.9)	−7.0
Warehouse/superstore	5 (2.1)	5 (7.3)	−5.2
Grocery	36 (15.2)	14 (20.3)	−5.1
Small store	157 (66.2)	29 (42.0)	24.2
Drug store	18 (7.6)	10 (14.5)	−6.9
**Percentage of Hispanic population in census tract**			
0.76%–4.11% (1st tertile)	105 (21.3)	29 (21.6)	−0.3
Supermarket	13 (12.4)	7 (24.1)	−11.7
Warehouse/superstore	1 (1.0)	1 (3.5)	−2.5
Grocery	19 (18.1)	12 (41.4)	−23.3
Small store	65 (61.9)	6 (20.7)	41.2
Drug store	7 (6.7)	3 (10.3)	−3.6
4.17%–6.75% (2nd tertile)	174 (35.3)	37 (27.6)	7.7
Supermarket	21 (12.1)	7 (18.9)	−6.8
Warehouse/superstore	1 (0.6)	1 (2.7)	−2.1
Grocery	35 (20.1)	6 (16.2)	3.9
Small store	98 (56.3)	17 (46.0)	10.3
Drug store	19 (10.9)	6 (16.2)	−5.3
6.78%–33.87% (3rd tertile)	214 (43.4)	68 (50.8)	−7.4
Supermarket	24 (11.2)	9 (13.2)	−2.0
Warehouse/superstore	5 (2.3)	5 (7.4)	−5.1
Grocery	32 (15.0)	11 (16.2)	−1.2
Small store	135 (63.1)	35 (51.5)	11.6
Drug store	18 (8.4)	8 (11.8)	−3.4
**Percentage of non-Hispanic Black or Hispanic population in census tract**			
0.79%–6.31% (1st tertile)	85 (17.2)	28 (20.9)	−3.7
Supermarket	19 (22.4)	7 (25.0)	−2.6
Warehouse/superstore	0	0	0
Grocery	14 (16.5)	8 (28.6)	−12.1
Small store	44 (51.8)	8 (28.6)	23.2
Drug store	8 (9.4)	5 (17.9)	−8.5
6.34%–15.15% (2nd tertile)	161 (32.7)	37 (27.6)	5.1
Supermarket	18 (11.2)	6 (16.2)	−5.0
Warehouse/superstore	2 (1.2)	2 (5.4)	−4.2
Grocery	33 (20.5)	6 (16.2)	4.3
Small store	90 (55.9)	20 (54.1)	1.8
Drug store	18 (11.2)	3 (8.1)	3.1
15.31%–50.22% (3rd tertile)	247 (50.1)	69 (51.5)	−1.4
Supermarket	21 (8.5)	10 (14.5)	−6.0
Warehouse/superstore	5 (2.0)	5 (7.3)	−5.3
Grocery	39 (15.8)	15 (21.7)	−5.9
Small store	164 (66.4)	30 (43.5)	22.9
Drug store	18 (7.3)	9 (13.0)	−5.7

a Stores defined as supermarkets must sell fresh meat, have 4 or more cash registers, and have at least 2 of the following staffed service counters: butcher, bakery, or deli. Stores defined as warehouses/superstores, such as Walmart and Costco, carry a wide array of products, usually including clothing, household items, and grocery items. Grocery stores must sell fresh meat and otherwise fail to meet the criteria for the supermarket or warehouse/superstore. Small stores do not have a butcher or fresh meat service counter and include establishments such as convenience stores, discount stores, and gas stations. Drug stores sell prescription and over-the-counter medications as well as other merchandise, including food and beverages.

b Differences in store type distributions are unweighted and provide justification for the use of postestimation weights in the primary analysis so that estimates reflect the store type distribution of all Seattle stores in each neighborhood tertile group.

c Tertiles are calculated from all census tracts in Seattle.

We used a categorized food permit database to define our store sampling frame. The database included all permitted food establishments in King County (includes Seattle) based on 2015 food permit records provided by Public Health–Seattle and King County and categorized by the University of Washington Urban Form Lab (20). We excluded all stores outside of Seattle, stores with duplicate permits, and restaurants.

**Figure 1:**
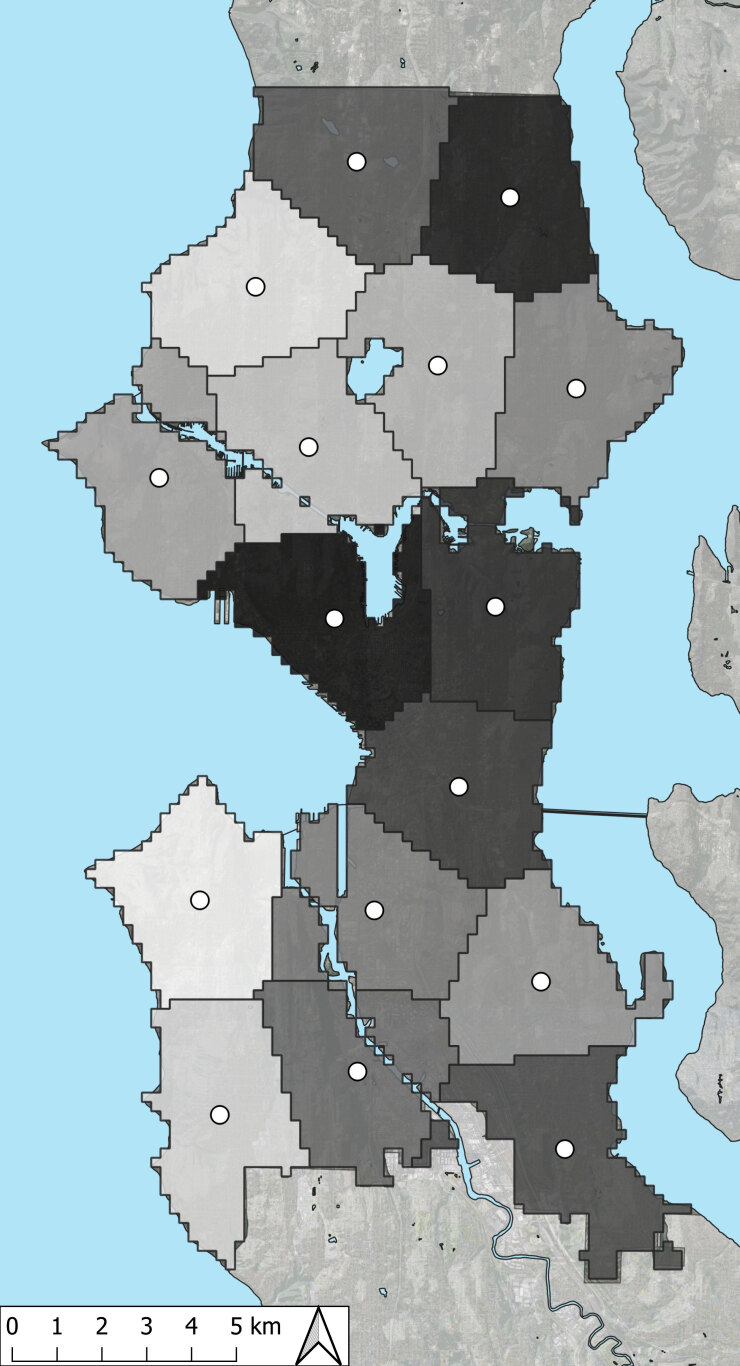
City of Seattle divided into 16 equal-sized areas used to select a geographically balanced sample for the study on price and availability of healthy foods in Seattle, Washington, neighborhoods, 2018.

To achieve a geographically balanced sample, we mapped all Seattle food retail establishments and divided the map into 16 equal-sized areas using the *spsample* function within the R package *spcosa* (21) (Figure 1). We calculated the centroid of each area and ordered the stores by distance from the centroid and store type. In each area, we selected a quota of stores from each store type as follows, prioritizing larger stores where most groceries are purchased: 1 supermarket, 2 grocery stores, 2 small stores, 1 drug store, and all warehouses/superstores. Some areas had fewer stores than the quota for that store type. We additionally worked with community liaisons to sample 10 more Black- or Hispanic-owned small stores; these stores were primarily located in 2 Seattle neighborhoods. We included these stores because the total sample was initially drawn to evaluate a sweetened beverage tax; the community advisory board anticipated a greater tax impact on these stores and requested their representation.

We used the 2016 US Census Boundary Files and 2012–2016 American Community Survey data to provide aggregate sociodemographic characteristics for all census tracts, including median household income and the proportion of the population across 5 racial and ethnic groups (22,23). We linked census tract data to the location of each store in the categorized food permit database and our final store sample. The resulting database was used to create postestimation weights for stores by neighborhood characteristics.

### Measurement tool

We developed the Seattle Healthy Food Survey as an abbreviated adaptation of the Nutrition Environment Measures Survey for Corner Stores (NEMS-CS) (8). The NEMS suite is among the most widely used tools for retail healthy food assessment. However, the tools are time-consuming to implement, and we aimed to create a shorter tool to reduce survey time and sample more stores. We chose to adapt and validate against the NEMS corner store version instead of the grocery store version because the former is more comprehensive, and we had many small stores in our sample. The NEMS-CS measures availability, price, and quality of food items within 13 categories: fresh and frozen/canned fruit and vegetables, milk, ground beef, hot dogs, frozen dinners, baked goods, beverages, bread, baked chips, and cereal. To select the food items for the Seattle Healthy Food Survey, we incorporated input from community partners, including the community advisory board, and leaders of local food access programs. We prioritized healthy foods that we anticipated appearing in our sample. Therefore, we added canned or dry beans, white or brown rice, eggs, and onions to the NEMS-CS and removed hot dogs, frozen dinners, baked goods, baked chips, and canned and frozen fruit and vegetables. We retained ground meat as the only meat item because the NEMS-CS includes only ground meat, and community partners thought this item would be available across store types. The final survey measured the availability and prices of 19 food items across these categories: fresh fruit, fresh vegetables, grains, protein, and milk (https://nutr.uw.edu/cphn/seattledrinktax/supplemental-materials).

**Figure 2:**
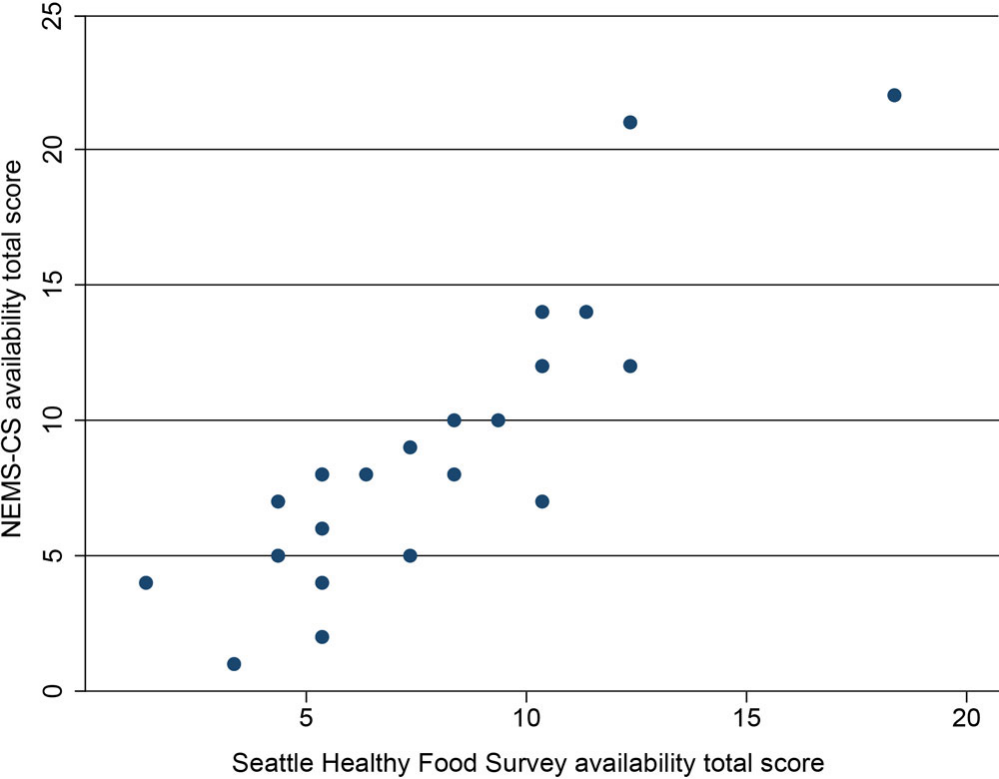
Scatterplot of Seattle Healthy Food Survey availability total score versus NEMS-CS availability total score, study on the price and availability of healthy foods in Seattle, Washington, neighborhoods, 2018. The Seattle Healthy Food Survey collects price and availability for 19 individual healthy food items within the categories of fruit, vegetables, grains, proteins, and milk. The NEMS-CS healthy food scoring algorithm was used to calculate total scores for the Seattle Healthy Food Survey. Abbreviation: NEMS-CS, Nutrition Environment Measures Survey for Convenience Stores.

To test the criterion validity of our measurement tool, we surveyed all food stores in 2 Seattle neighborhoods with suspected limited availability of healthy foods. Trained data collectors surveyed 23 food stores using both the Seattle Healthy Food Survey and NEMS-CS. The sample comprised 18 small stores, 3 grocery stores, 1 supermarket, and 1 drug store. We calculated the Pearson correlation coefficient of the healthy food availability scores for each survey across all stores. The Seattle Healthy Food Survey was strongly correlated with the NEMS-CS overall (*r* = 0.875) and within each store type in the sample (small stores *r* = 0.814; grocery stores *r* = 0.929). Figure 2 illustrates the relationship between the total healthy food availability scores produced by each measurement tool across all stores.

Data collectors attended one 6-hour training and practiced data collection in the field with the Seattle Healthy Food Survey until achieving an overall average 90% raw agreement on all responses.

### Dependent variables

The primary outcomes of interest were healthy food availability and healthy food prices across food store types in Seattle.

**Healthy food availability score.** Each healthy food item on the Seattle Healthy Food Survey that was available in a store received at least 1 point. Healthier items received more points than their less-healthy counterparts of the same food type. For example, 100% whole-grain bread was worth 2 points and white bread 1 point (https://nutr.uw.edu/cphn/seattledrinktax/supplemental-materials). An item was considered available if it was present in the specified size on the survey. If items were not available in the specific size and form, similar items were measured (eg, dried instead of canned beans). For each store, we summed all points for each food item to produce an overall availability score ranging from 0 to 25. A higher score indicated greater availability of healthy foods.

**Healthy food price.** We calculated the mean price in US dollars per pound or gallon for fruits, vegetables, grains, protein, and milk. We considered the prices for protein items separately because the prices of the items varied dramatically, and stores often had only 1 type of protein available. We used the nonsale price of the least-expensive available item to calculate the mean prices.

### Independent variables

The primary independent variables were neighborhood-level measures of income and racial and ethnic composition linked to the store(s) within the census tract.

**Neighborhood median household income.** We used the 2012–2016 American Community Survey 5-year estimates of census tract–level median household income to define neighborhood median household income (22). Across all census tracts in Seattle, we calculated tertiles of median household income to represent low-, middle-, and high-income neighborhoods in Seattle. We linked each store’s location to its census tract and stratified our store sample based on the tertiles of Seattle neighborhood median household income.

**Neighborhood racial and ethnic composition.** We used the same 2012–2016 American Community Survey data to determine the proportion of the population in each census tract who reported Black or African American race, and separately, the proportion who reported Hispanic ethnicity. Previous literature notes consistent disparities in food environments comparing neighborhoods with larger proportions of Black or Hispanic populations to neighborhoods with smaller proportions (2,5,9). We assessed these measures separately because the commercial histories of the food retail environment and neighborhood locations differ between Black and Hispanic communities because of Seattle’s history of racial restrictive covenants and redlining (24). In initial analyses, we created a combined measure, proportion non-Hispanic Black or Hispanic, to capture aspects of the food environment related to inequities that communities of color experience across Seattle. For the previously stated reasons, we considered this combined measure as a secondary analysis. We used all Seattle census tracts to calculate tertiles of the proportions of the population who were Black and who were Hispanic. We stratified the stores on each set of tertiles using each store’s census tract location. Tertile value ranges and the distribution of stores within each tertile are in Table 1.

### Statistical analysis

We described the mean and 95% CI for healthy food availability scores and price per pound or gallon within each store type. For the primary analysis, we estimated the mean and 95% CI for healthy food availability scores and price per pound or gallon by neighborhood median household income and racial and ethnic composition across all store types. We used Bonferroni-adjusted Wald tests to compare the mean availability scores and prices between tertiles. The reference groups were neighborhoods with the highest income, the smallest proportion of Black residents, and the smallest proportion of Hispanic residents. Because each test involves 2 comparisons, the Bonferroni adjustment mitigates the increased risk of a type I error by producing bounded *P* values that are twice as large as they would be in an unadjusted comparison. We considered significance at the α level of .05.

To ensure the primary results were representative of the makeup of store types in Seattle across neighborhood tertiles, we applied postestimation weights. The weights were based on store types because we had a database of all stores in Seattle, and store type is correlated with the healthfulness, availability, and price of foods sold (10,11,13). We created 3 sets of weights corresponding to each store’s census tract: 1 for neighborhood median household income, and 1 each for neighborhood proportion of Black and Hispanic residents. Weights adjusted the results to reflect the distribution of all store types within each tertile of neighborhood sociodemographic characteristics. We expected healthy food availability and prices to vary by store type, and similarly, that store type mix varied by neighborhood sociodemographic composition. Therefore, by using these weights, we aimed to capture both sources of variation and to produce results that reflected the landscape of healthy food access that residents experience in their neighborhood. We applied poststratification adjustments to the weights using poststratum identifiers to account for nesting of multiple stores within each census tract. In addition, we used a finite population correction factor that adjusts estimated variances to account for a sample that is large relative to the size of the population from which it is drawn. As appropriate, weights were adjusted for use in subpopulation estimates. We performed all analyses using Stata version 15.1 (StataCorp LLC).

## Results

Across Seattle census tracts, the 2012–2016 5-year estimate for median household income at the census tract level was $74,915 (range, $10,865–$159,652). The median census tract–level proportion of Black residents was 3.82% (range, 0%–40.0%), and for Hispanic residents, the median population proportion was 5.42% (range, 0.76%–33.87%). The categorized food permit database contained 493 food stores. We surveyed 27% (n = 134) of these food stores (https://nutr.uw.edu/cphn/seattledrinktax/supplemental-materials). Compared with the distribution of all stores in Seattle, our sample included relatively more supermarkets (17% vs 12%), warehouses/superstores (5% vs 1%), grocery stores (22% vs 17%), and drug stores (13% vs 9%), and fewer small stores (43% vs 61%) (Table 1).

### Healthy food availability and prices, by store type

Of the stores in our sample, 96% (n = 128) carried at least 1 healthy food item (Table 2). Of 25 total points, warehouses/superstores had the highest mean healthy food availability score (20.6), followed by supermarkets (19.0) and grocery stores (16.2). Drug stores (9.5) and small stores (6.1) had substantially lower mean healthy food availability compared with the larger store types. Drug stores had a higher mean availability score than small stores despite carrying no fruit, vegetables, or meat. This is largely due to the consistency in carrying products across all other food groups; all drug stores carried eggs, beans, milk, and grains. The availability of foods in small stores ranged widely; 67% (n = 39) carried milk, 62% (n = 36) carried grains, 50% (n = 29) carried fresh fruit, 45% (n = 26) carried proteins, and 22% (n = 13) carried fresh vegetables. Only 1 small store carried fresh meat.

**Table 2:** Average Healthy Food Availability Scores and Prices of Healthy Foods in Seattle, Washington, by Store Type, 2018^a^

Store type	Healthy food availability score^b^	Healthy food prices, $						
		Fruit per pound	Vegetables per pound	Grains per pound	Beans per pound	Eggs per pound	Meat per pound	Milk per gallon
	Mean score (95% CI), no. of stores							
Supermarket	19.00 (15.91–22.09), n = 23	1.58 (0.98–2.17), n = 22	1.69 (1.57–1.81), n = 21	2.41 (2.04–2.79), n = 23	1.24 (1.12–3.67), n = 19	1.34 (1.11–1.56), n = 18	5.78 (5.17–6.39), n = 18	3.41 (2.70–4.13), n = 20
Warehouse/ superstore	20.57 (17.86–23.29), n = 7	1.46 (0.77–2.16), n = 7	1.92 (1.19–2.66), n = 7	1.90 (1.71–2.09), n = 7	0.91 (0.77–1.05), n = 7	1.71 (0.58–2.84), n = 6	5.45 (4.69–6.21), n = 6	2.88 (2.26–3.51), n = 7
Grocery	16.21 (14.10–18.31), n = 29	1.99 (1.35–2.63), n = 26	1.97 (1.63–2.30), n = 28	2.39 (1.79–2.99), n = 26	1.57 (1.42–1.73), n = 23	2.12 (1.80–2.44), n = 25	4.95 (4.02–5.89), n = 20	4.17 (3.67–4.67), n = 25
Small store	6.09 (4.81–7.37), n = 58	2.77 (2.41–3.13), n = 29	2.35 (1.48–3.21), n = 13	2.29 (1.88–2.70), n = 36	2.11 (1.78–2.44), n = 24	2.72 (2.42–3.01), n = 28	3.99 (—), n = 1	5.18 (4.68–5.68), n = 39
Drug store	9.47 (8.76–10.18), n = 17	—	—	2.59 (1.73–3.44), n = 17	1.71 (1.52–1.89), n = 10	1.76 (1.47–2.04), n = 17	—	3.34 (3.10–3.59), n = 17

Abbreviation: — , not applicable.

a Stores defined as supermarkets must sell fresh meat, have 4 or more cash registers, and have at least 2 of the following staffed service counters: butcher, bakery, or deli. Stores defined as warehouses/superstores, such as Walmart and Costco, carry a wide array of products, usually including clothing, household items, and grocery items. Grocery stores must sell fresh meat and otherwise fail to meet the criteria for the supermarket or warehouse/superstore. Small stores do not have a butcher or fresh meat service counter and include establishments such as convenience stores, discount stores, and gas stations. Drug stores sell prescription and over-the-counter medications as well as other merchandise, including food and beverages.

b Score ranges from 0–25 points, with a higher score indicating greater availability. Fruit includes apples, oranges, and bananas. Vegetables include broccoli, carrots, green lettuce, tomatoes, and yellow onions. Grains include 100% whole wheat bread, white bread, Frosted Flakes cereal, Original Cheerios cereal, and rice (white or brown). Beans include canned black, kidney, and garbanzo beans. Meat includes lean fresh ground meat. Milk includes fat-free milk, 1% milk, 2% milk, and whole milk; the mean milk price was drawn from fat-free milk if available, then 1% milk, then 2% milk, then whole milk.

For most food categories, prices were generally lower in larger stores (supermarkets and warehouses/superstores) than in smaller stores (grocery, small, and drug stores). While grocery stores had a lower mean price for meat compared with supermarkets and warehouses/superstores, these differences were not significant. Small stores had the highest average prices compared with other store types for nearly all food categories except grains. For milk, drug stores had a similarly lower price compared with supermarkets, while small stores had the highest prices, on average. Within each store type, mean prices tended to be highest for meat and milk and lower for fruits and vegetables.

### Healthy food availability and prices, by neighborhood sociodemographic characteristics

Table 3 presents the weighted healthy food availability scores and prices by tertiles of neighborhood (census tract) sociodemographic characteristics, across all surveyed stores. Across neighborhood median household income groups, lower-income neighborhoods had lower healthy food availability scores compared with higher-income neighborhoods (8.06 [95% CI, 7.04–9.07] vs 12.40 [95% CI, 10.63–14.17], *P < .*001). When comparing across tertiles of neighborhood proportion of Black residents, those with the largest proportion of Black residents had lower healthy food availability scores than neighborhoods with the smallest proportion (8.88 [95% CI, 7.79–9.98] vs 12.32 [95% CI, 10.51–14.14], *P = .*003). Across tertiles of neighborhood proportion of Hispanic residents, healthy food availability scores were similar (highest tertile: 9.01 [95% CI, 7.95–10.07], lowest tertile: 10.63 [95% CI, 8.01–13.25], *P = .*52).

**Table 3:** Average Healthy Food Availability Score and Price Per Pound of Healthy Foods in Seattle According to Neighborhood Income and Proportions of the Population Who Are Black and Hispanic, Seattle, Washington, 2018

Characteristic and tertile (no. of stores)	Healthy food availability score^a^, weighted^b^ (N = 134)	Healthy food prices, $ (no. of stores), weighted^b^						
		Fruit per pound (n = 84)	Vegetables per pound (n = 69)	Grains per pound (n = 109)	Beans per pound (n = 76)	Eggs per pound (n = 90)	Meat per pound (n = 43)	Milk per gallon (n = 108)
	Mean (95% CI)							
**Median household income in census tract**								
1st tertile: $10,865–$65,772 (n = 62)	8.06 (7.04–9.07)	2.45 (2.04–2.87)	1.63 (1.40–1.87)	2.21 (1.89–1.52)	1.73 (1.52–1.95)	2.06 (1.90–2.23)	4.95 (4.21–5.70)	4.45 (4.05–4.84)
*P* value^c^	<.001	.39	.18	.06	.97	.07	.17	.99
2nd tertile: $65,781–$90,688 (n = 46)	10.75 (9.44–12.07)	2.37 (2.00–2.75)	2.36 (1.98–2.74)	2.19 (1.83–2.54)	1.75 (1.51–2.00)	2.27 (2.02–2.52)	5.16 (4.49–5.84)	4.61 (4.11–5.11)
*P* value^c^	.28	.56	.83	.06	.99	.76	.34	.99
3rd tertile: $90,855–$159,652 (n = 26)	12.40 (10.63–14.17)	2.10 (1.76–2.44)	2.10 (1.61–2.59)	2.81 (2.38–3.24)	1.84 (1.62–2.06)	2.45 (2.13–2.76)	5.82 (5.16–6.48)	4.52 (4.12–4.92)
	1 [Reference]							
**Percentage of Black population in census tract **								
1st tertile: 0%–1.41% (n = 28)	12.32 (10.51–14.14)	2.19 (1.85–2.53)	2.29 (1.77–2.81)	2.78 (2.35–3.22)	1.73 (1.44–2.02)	2.52 (2.29–2.75)	5.84 (5.23–6.46)	4.51 (4.05–4.97)
	1 [Reference]							
2nd tertile: 1.43%–7.17% (n = 37)	10.03 (8.87–11.20)	2.42 (2.06–2.78)	2.19 (1.77–2.60)	2.47 (2.10–2.83)	1.91 (1.70–2.12)	2.29 (2.07–2.50)	5.80 (5.03–6.56)	4.40 (4.09–4.70)
*P* value^c^	.08	.70	.99	.54	.64	.29	.99	.99
3rd tertile: 7.31%–40.01% (n = 69)	8.88 (7.79–9.98)	2.33 (1.94–2.72)	1.77 (1.44–2.10)	2.14 (1.83–2.45)	1.70 (1.49–1.91)	2.05 (1.82–2.28)	4.67 (4.09–5.25)	4.64 (4.16–5.12)
*P* value^c^	.003	.99	.20	.04	.99	.009	.01	.99
**Percentage of Hispanic population in census tract **								
1st tertile: 0.76%–4.11% (n = 29)	10.63 (8.01–13.25)	2.33 (1.95–2.70)	1.88 (1.63–2.13)	2.76 (1.99–3.53)	1.67 (1.45–1.90)	2.38 (1.67–3.09)	5.55 (4.88–6.22)	4.54 (3.78–5.29)
	1 [Reference]							
2nd tertile: 4.17%–6.75% (n = 37)	10.79 (9.48–12.09)	2.34 (1.95–2.73)	2.10 (1.54–2.65)	2.67 (2.19–3.15)	1.73 (1.52–1.94)	2.31 (2.09–2.52)	5.50 (4.52–6.48)	4.24 (3.84–4.65)
*P* value^c^	.99	.99	.96	.99	.99	.99	.99	.99
3rd tertile: 6.78%–33.87% (n = 68)	9.01 (7.95–10.07)	2.52 (2.09–2.95)	2.12 (1.78–2.46)	2.12 (1.85–2.40)	1.80 (1.57–2.02)	2.19 (1.98–2.41)	4.78 (4.17–5.39)	4.71 (4.32–5.09)
*P* value^c^	.52	.98	.53	.26	.89	.99	.19	.99

a Score ranges from 0–25 points, with a higher score indicating greater availability. Fruit includes apples, oranges, and bananas. Vegetables include broccoli, carrots, green lettuce, tomatoes, and yellow onions. Grains include 100% whole wheat bread, white bread, Frosted Flakes cereal, Original Cheerios cereal, and rice (white or brown). Beans include canned black, kidney, and garbanzo beans. Meat includes lean fresh ground meat. Milk includes fat-free milk, 1% milk, 2% milk, and whole milk; the mean milk price was drawn from fat-free milk if available, then 1% milk, then 2% milk, then whole milk.

b Postestimation weights and post-stratification adjusted results to the city-wide distribution of store types within tertiles of household income, percentage of the population that is Black, and percentage of the population that is Hispanic. Finite population correction and, as appropriate, subpopulation sizes were adjusted for. Tertiles were computed from all census tracts in Seattle (N = 135), using 2012–2016 American Community Survey data (22).

c Bonferroni-adjusted *P* values for an Adjusted Wald Test comparing means in each tertile interval to the reference tertile interval (eg, ≥$90,855 for median household income). Because each test involves 2 comparisons, the adjustment produces bounded *P* values that were twice as high as would be expected in an unadjusted comparison.

Healthy food prices generally did not differ across tertiles of neighborhood median household income. Mean prices per pound of vegetables, grains, eggs, and meat appeared lower in lower-income neighborhoods, but these differences were not significant. Prices of milk and beans were consistent across neighborhood income groups. Similarly, mean prices were generally comparable across tertiles of neighborhood proportion of Black residents and neighborhood proportion of Hispanic residents. A few exceptions were the mean prices of grains, eggs, and meat, which were lower in neighborhoods with the largest proportion of Black residents compared with neighborhoods with the smallest proportion. Prices did not differ between the intermediate tertile group and the smallest tertile group for neighborhood proportion of Black residents.

Across tertiles of neighborhood proportion of non-Hispanic Black or Hispanic residents, results followed a similar pattern to neighborhoods defined by proportion of Black residents (https://nutr.uw.edu/cphn/seattledrinktax/supplemental-materials).

## Discussion

In this large, representative sample of food stores in Seattle, we found evidence of sociodemographic disparities in healthy food availability. We did not find evidence of systematic differences in healthy food prices by these neighborhood characteristics. Altogether, our findings provide context to the broader hypothesis that limited availability and affordability in the local food environment may contribute to poor diet quality and health outcomes in under-resourced neighborhoods.

Differential availability of healthy food based on income and racial and ethnic composition is documented in other urban settings. Previous studies have evaluated cities’ food environments — including Portland, Honolulu, Kansas City, and Baltimore — and found similar inequities in healthy food availability by store type and proximity (12,14,15). Our study extends the literature by investigating in-store healthy food availability in a representative sample of Seattle stores that accounts for store type variations across neighborhood sociodemographic contexts. Our findings reinforce that cities with histories of racial segregation, gentrification, and rapid development, such as Seattle, possess systemic barriers to healthy food access (14,15,25). Our results suggest that lower-income and more racially diverse neighborhoods have less healthy food availability across a variety of items rather than one type of food. The systematic difference in healthy food availability may contribute to diet quality disparities in populations with lower socioeconomic status and in Black populations. Moreover, individuals with lower income may be particularly vulnerable to limited availability in their neighborhood because regular travel to areas with greater availability requires additional resources.

Price of food is a barrier to healthy dietary behaviors (26,27). Our findings that healthy food prices did not differ based on neighborhood-level income suggest that healthy food is more expensive, relative to household income, for economically disadvantaged groups compared with more advantaged groups in Seattle. Economic theory supports that food purchases are income-sensitive; individuals with lower socioeconomic status are more sensitive to food prices compared with those with higher socioeconomic status (28). Other studies have found that healthy diets are often financially out of reach for low-income individuals and those living in poverty, even when receiving government assistance (26,29). Research has also found that higher socioeconomic status is associated with better diet and higher dietary cost (18,30).

Our study has limitations. Although we surveyed a large sample of stores, we did not survey all stores. We created postestimation weights using the 2015 distribution of stores, which may not reflect changes after 2015. Additionally, individuals do not always shop for food at stores most proximal to their home (1,11); limits exist with regard to inferring food access based on stores within a given neighborhood. Furthermore, we did not survey several popular stores such as Whole Foods, Trader Joe’s, and PCC because our original sample was drawn to evaluate a sweetened beverage tax, and these stores tend to sell few sugary beverages. The sample size, and thus the statistical power, for the analyses on price of lower-frequency items (eg, meat) was low. Finally, although our survey performed well compared with the gold-standard NEMS-CS, we measured only 3 fresh fruits and 5 fresh vegetables and may have missed other healthy foods (eg, more culturally relevant heathy foods). We did not measure canned or frozen fruit or vegetables, which are measured in the NEMS-CS and may have been available. By validating against the NEMS-CS, we are reassured that our shorter tool is able to distinguish relatively healthier versus less healthy food availability overall. It is notable that the tertiles herein are specific to Seattle and thus reflect smaller Black and Hispanic populations and higher income than many other metropolitan areas in the US.

To achieve an equitable, healthy food environment, healthy food must be accessible, available, affordable, and culturally appropriate (1,15). Our study developed and validated an abbreviated survey tool to conduct assessments in a large, geographically representative sample of Seattle stores to examine 2 dimensions of the food environment: availability and affordability. By collecting in-store food availability and price data across a variety of store types and weighting estimates to the store landscape, our study provides a multilevel analysis of Seattle’s retail food environment. Our findings suggest that policies that increase community resources and availability of healthy food in the local food environment could benefit neighborhoods with lower incomes and larger proportions of Black residents. Local assessments of the food retail environment can help policy makers and public health practitioners implement tailored strategies to comprehensively improve healthy food access.
